# 肺微乳头腺癌病理学及分子学特征研究进展

**DOI:** 10.3779/j.issn.1009-3419.2020.102.37

**Published:** 2020-11-20

**Authors:** 佳凤 梁, 琼 吴, 胜林 马, 仕蓉 张

**Affiliations:** 1 310006 杭州，浙江大学医学院附属杭州市第一人民医院转化医学中心，浙江省临床癌症药理毒理研究重点实验室 Translational Medicine Research Center, Key Laboratory of Clinical Cancer Pharmacology and Toxicology Research of Zhejiang Province, Hangzhou 310006, China; 2 310006 杭州，浙江大学医学院附属杭州市第一人民医院肿瘤科 Department of Oncology, Affiliated Hangzhou First People's Hospital, Zhejiang University School of Medicine, Hangzhou 310006, China

**Keywords:** 微乳头腺癌, 转移, 复发, 驱动基因, 免疫微环境, Micropapillary adenocarcinoma, Metastasis, Recurrence, Driver gene, Immune microenvironment

## Abstract

肺微乳头腺癌作为高级别肺腺癌，具频发转移、淋巴结浸润、复发率高和总体生存率低的临床特征。该亚型肿瘤中存在特征致癌因子通路的激活和肿瘤免疫微环境的建立。本文拟对近年来微乳头腺癌的病理学表现及分子学特征研究进展作一综述，旨在加深对微乳头型病变的认识，进而为制定特异性治疗策略奠定基础。

## Abstract (en)

Lung micropapillary adenocarcinoma is characterized by frequent metastasis, lymph node infiltration, high recurrence rate and low overall survival rate as a high-grade lung adenocarcinoma. Special oncogenic pathway is activated and immune microenvironment is established in this subtype of tumor. This article reviews the Pathological phenomena and molecular features of micropapillary adenocarcinoma studied in recent years, aiming to deepen the understanding of micropapillary lesions and lay the foundation for formulating specific treatment strategies.

2018年世界卫生组织公布，肺癌已成为发病率和死亡率均占首位的癌种^[1]^。自2009年吉非替尼被应用于表皮生长因子受体(epidermal growth factor receptor, *EGFR*)驱动基因阳性肺腺癌(lung adenocarcinoma, LADC)人群的一线治疗^[2]^，2015年免疫治疗药物Nivolumab运用于晚期非小细胞肺癌患者的二线治疗^[3]^，两种精准治疗方案的快速发展使患者的生存期及生活质量获得了一定程度的改善。

肺微乳头型腺癌(lung micropapillary adenocarcinoma, LMPC)是一种高级别低分化的LADC亚型，临床数据显示该类患者生存预后较其他亚型患者差，最佳治疗方案的制定可提高目标人群的生存获益。本文将对近年来MPC的病理学表现及分子学特征研究进展作一综述以完善常规手术、放化疗方案制定以及指导靶向治疗及免疫治疗在此类LADC亚型中的应用。

## 1 LMPC

肺癌的组织学亚型分类及基因型分类在对肺癌的治疗过程中起到了治疗指导及预后判断的作用。根据世界卫生组织规定，肺癌的组织学分型主要包括鳞癌、腺癌、大细胞癌、腺鳞癌、小细胞癌等，腺癌可进一步分为原位腺癌/浸润前病变，微浸润性腺癌和浸润性腺癌这三类，其中浸润性腺癌可表现出多种组织学生长模式，包括贴壁性、乳头状、腺泡状、实性腺癌以及微乳头状。2011年，微乳头型被新纳入浸润性腺癌分型且已有部分相关临床预后报告，但目前尚无较全面的遗传学相关研究^[4]^。

### 1.1 LMPC的流行病学

肿瘤病理学检验结果显示在实际临床诊断中只有约20%的LADC由单一病理亚型组成^[5]^，而大多数LADC是两种或两种以上不同组织学模式的混合肿瘤，不同组织学比例组合模式可有不同的流行病学结果及相应的生存预后表现。在病理学检验过程中，因存在被检验肿瘤患者人群差异、病理医师判定差异、样本处理差异，各研究报道的微乳头成分在LADC中发生比例相差较大。有研究^[6]^显示，在525例浸润性LADC患者中有114例患者肿瘤的微乳头成分 > 5%，115例含有微量微乳头成分(1%-5%)，即43.6%的浸润性LADC患者中具微乳头成分生长模式，但另有研究^[7]^结果与此数据相差较大，该研究显示在浸润性LADC患者中27.5%的患者含有微乳头成分; 而微乳头成分占主要比例的LADC发生比例较少，约为5%^[8, 9]^。由此可见含部分微乳头生长模式在浸润性LADC中常发。

### 1.2 LMPC的临床表现

LMPC的临床表现与其余的肺癌无明显差别，可出现咳嗽、胸闷胸痛、消瘦等症状以及部分患者可出现淋巴结肿大体征。

### 1.3 LMPC的影像学及病理学诊断

临床影像学相关研究显示，在肿瘤分期大于Ⅰ期、大小≥2.5 cm、实体肿瘤组织块、正电子发射计算机断层显像(positron emission tomography-computed tomography, PET-CT)最大标准化摄取值≥7，这些条件下，可预测微乳头亚型或实体亚型的存在^[10]^。而在高分辨率CT下的结节中，含微乳头成分的LADC相较于磨玻璃样和半实性，更易表现为实性结节^[11]^。

LADC中微乳头生长模式的组织病理学特征表现为从肿瘤肺泡间隙或结缔组织所包裹的间隙中分离出小乳头状簇，这些簇几乎没有或没有中央纤维血管核心，且细胞极性消失，并与基质脱离，这一无序的漂浮在组织间液的细胞团块表现可能有助于该亚型的转移^[12, 13]^。

在LMPC中可出现部分特殊病理学表现，包括肿瘤通过气隙扩散(tumor spread through air space, STAS)形成单细胞转移灶或肿瘤岛(指存在于各肺泡腔内的大量肿瘤组织，且这些组织间存在连接，该表型与患者生存预后和复发相关)以及易出现肿瘤淋巴结微转移(在淋巴结中存在单个肿瘤细胞或肿瘤细胞块最大径线≤0.2 mm)等，这些因素与肿瘤复发以及干扰预后相关^[14-16]^。

### 1.4 LMPC的治疗及预后

目前LMPC的治疗模式依据LADC的治疗方案展开，主要包括了手术治疗、化疗、放疗以及驱动基因阳性下的靶向治疗和免疫标志物达标下的免疫治疗。而在上述治疗手段下，微乳头亚型患者受益较其他亚型受限。在肺癌的各种组织学分型中，以微乳头型和实性为主的腺癌发生胸腔外复发概率高，且总生存率相对其他组织学分型较低^[17]^。相较于不含微乳头成分的肿瘤患者而言，即使是最小量微乳头型的患者(微乳头含量小于整个肿瘤的5%且大于1%)也会出现总生存预后不良^[6]^，且复发风险率升高^[18]^。一项在澳大利亚开展的研究^[19]^发现，LADC患者无论处于Ⅰ期、Ⅱ期、Ⅲ期中任何阶段，LMPC患者在5种肺癌分型中总生存期预后均较差。

有多项研究结果显示此类患者预后较差的原因与肿瘤复发、转移、淋巴结浸润及治疗抵抗等因素相关。有研究^[20]^表明Ⅰ期LADC患者有20.1%的复发率，除年龄大、肿瘤组织侵犯脏层胸膜表面外，高级别LADC即微乳头和实性LADC也是复发的预测因素。即使是 < 3 cm且淋巴结阴性的LADC患者群体，若组织学表现为微乳头/实性占优势，则该患者的复发风险较不占优势者高^[21]^。对于LADC肿瘤组织≤2 cm的患者，亚组分析发现组织学为微乳头型的患者更有可能发生淋巴结转移^[22]^。

### 1.5 LMPC的治疗方案探索

有关腺癌亚分型治疗研究发现，当Ⅰ期和IIa期患者进行立体定向放射治疗，微乳头和实性组织肿瘤表现出局部区域和转移肿瘤进展较其余亚型更快^[23]^，故可推测加大放疗剂量、添加辅助治疗手段或更换为手术治疗的方案对该亚型患者更有益。而在适合手术治疗的患者中也出现了治疗效果不佳情况，在肺癌手术切除后复发的患者中，肿瘤组织分型表现为微乳头性和实性的患者生存期预后较差，为改善LMPC的治疗情况，术式的选择及辅助治疗方案的加入有增加患者获益的可能^[24]^。在微乳头病变 > 5%的Ⅰ期患者中，楔形或节段切除5年复发率明显高于肺叶切除，提示即使是早期肺肿瘤，微乳头病变也易通过肺组织转移而非仅发生浸润病变，这也表明微乳头肿瘤需要较其他亚型更积极的治疗方案^[25]^。目前Ⅰ期LADC患者在手术治疗后并不常规推荐辅助治疗，但近年来已有多项研究^[18, 26-28]^显示Ib期LMPC可受益于术后辅助化疗，包括降低复发率及延长无病生存期; 此外，近日发表的一项对Ia期LMPC患者研究^[29]^表明术后辅助化疗可延长患者无进展生存期及总生存期，由此可见Ⅰ期LMPC患者可能不同于其他亚型，需强调术后辅助化疗的应用。而对手术联合辅助放疗的研究发现，此方案并不能改善Ⅰ期-Ⅲ期LMPC患者的预后，我们可推测微乳头亚型可能对放疗具高耐受性或常发生射野外的早期转移^[30]^。上述研究结果均为回顾性分析，且未考虑术式类型及辅助方案的一致性，因此前瞻性大样本试验的验证值得我们期待。

此外值得我们关注的是，晚期LADC患者预后情况与早期不同，MPC对化疗(铂类)的反应较鳞状腺癌、腺泡腺癌等低级别LADC更佳，患者总生存期相对较长^[31]^，研究者推测出现该现象的原因与不同亚型瘤内异质性相关，但因该研究主要针对晚期肺癌患者，采集到的样本量有限，且为单中心回顾性研究，该结果及相应机制需进一步探索发现。

### 1.6 LMPC的分子学特征

近年来精准化治疗取得了良好成果，因此对肿瘤组织中组织学异质性与分子学差异关系的研究可为患者治疗新方案的确立奠定基础。有研究^[8]^通过显微切割肿瘤组织分析肿瘤组织学亚型对驱动基因异质性表达的影响，发现间变性淋巴瘤激酶(anaplastic lymphoma kinase, *ALK*)融合与微乳头状模式呈正相关，从而可推测克唑替尼、色瑞替尼等ALK靶向药物可能使微乳头结构含量较高患者获益。另有研究^[7]^通过对微乳头占主要型的患者样本分析发现，该类患者存在*EGFR*的驱动基因突变富集，回顾性研究^[32]^也证实此类患者可受益于酪氨酸酶抑制剂(tyrosine kinase inhibitors, TKIs)治疗，但这富集并未在所有相关研究中均显示出统计学差异^[33]^。鼠类肉瘤病毒癌基因同源物B1(V-raf murine sarcoma viral oncogene homolog B1, *BRAF*)V600E的突变也被证实与微乳头成分存在相关性，较其他亚型而言微乳头亚型中更易出现*BRAF*突变，由此我们可推测*BRAF*突变可能可推动了微乳头模式的分化^[9, 34]^。MPC所表现出的上述独特基因改变(表 1)，可为其靶向治疗方案的开发奠定基础。

**表 1:** 肺微乳头型腺癌遗传学变异 Genetic variation of lung micropapillary adenocarcinoma(LMPC)

Patients’pathological type	Sampling methods	Numbers	Micropapillary component ratio in defined LMPC patients	Defined LMPC patients’ number	Specific genetic variation	The percentage of variated patients in LMPC patients	The percentage of related variated patients in their lung cancer subtypes	Whether variation correlated with micropapillary components	Testing methods	Ref
Primary LADC	Surgery	629	Predominant	215 (3.3%)	*ALK* fusion	3(14.3%)	0.0%-6.5%	Correlated	AmoyDx *EML4*-*ALK* fusion gene detection kit tested initially and verified by sequencing	^[8]^
					*EGFR* mutation	8 (38.1%)	16.7%-80.0%	Not correlated	AmoyDx *EGFR* 29 mutation detection kit tested initially and verified by sequencing	
Invasive LADC	Surgery	211	Included	585 (27.5%)	*EGFR* mutation	53 (91.4%)	59.4%	Correlated	HRMA tested initially and detected by ARMS	^[7]^
Invasive LADC	Surgery	425	Predominant	25 (6.2%)	*ALK* rearrangement	0 (0.0%)	0.0%-2.8%	Not correlated	IHC tested initially and verified by FISH	^[9]^
					*EGFR* mutation (Exon 18-21)	8 (32.0%)	9.4%-23.3%	Correlated	Sanger sequence	
					*Kras* mutation (Exon 1)	9 (36.0%)	32.4%-40.9%	Not correlated		
					*BRAF* mutation (Exon 15)	2 (8.0%)	0.0%-5.4%	Not correlated		
Primary LADC	Surgery	1, 046	Predominant or second predominant	unknown	*BRAF* V600E	80.0%	unknown	BRAF genes in micropapillary components are mostly V600E mutations	HRMA tested initially and detected by ARMS	^[34]^
LADC: lung adenocarcinoma; EGFR: epithelial growth factor receptor; ALK: anaplastic lymphoma kinase; Kras: Kirsten ratsarcoma viral oncogene homolog; BRAF: V-raf murine sarcoma viral oncogene homolog B1; HRMA: high resolution melting analysis; ARMS: amplification refractory mutation system; FISH: fluorescence in situ hybridization.										

部分与肺微乳头成分相关肿瘤蛋白已被验证。有研究^[35]^表明神经源性位点切迹同源蛋白1(neurogenic locus Notch homolog protein 1, Notch-1)的表达与肿瘤复发相关，通过免疫组化(immunohistochemistry, IHC)检测发现在肺癌各类亚型中，实性腺癌Notch-1表达呈阴性，而微乳头状腺癌中表达含量相对较高，类似的微乳头相关特异性表达蛋白包括vimentin、Napsin、CD10等^[30]^。基于上述发现提示，通过聚焦LMPC中特异性的通路变化，可为开发抑制剂、抗体类药物等治疗方案与传统治疗手段结合新策略提供指导。此外，一项研究^[36]^发现免疫治疗热点靶标PD-L1表达的异质性也与微乳头病变相关，但其明确的表达关系及治疗方案制定仍需进一步深入研究。

## 2 其他肿瘤MPC

LADC并不是唯一的具微乳头状结构的肿瘤。微乳头状结构最早报道于1993年，见于乳腺浸润性导管腺癌中^[37]^。其后在多种肿瘤中均可见，包括唾液腺癌、涎腺导管癌、甲状腺癌、乳腺癌、胸膜间质瘤、胃癌、胰腺癌、结直肠癌、尿路上皮癌、子宫颈癌等^[38-47]^。通过分析其他肿瘤微乳头亚型腺癌的病理学及分子学特征可为LMPC相关研究提供一些启示。

### 2.1 MPC的预后及病理学表现

与LMPC恶性程度较高相似，微乳头亚型的存在除在甲状腺微乳头状癌患者中表现为死亡率低外，其余肿瘤类型如乳腺癌、胃癌、结直肠癌、尿路上皮癌、宫颈癌等均表现出高度侵袭性或对预后具不良影响，且常表现出淋巴结转移特征。有研究^[45]^对大肠微乳头状腺癌的干细胞表型进行了鉴定，结果显示该组织学癌种中癌症干细胞标记物高表达，故而可对微乳头状腺癌高恶性程度做出部分相关机制解释说明。

### 2.2 MPC的分子学特征

为进一步明确肿瘤遗传学改变以追本溯源寻求治疗方案，研究者们对部分肿瘤的微乳头状癌分型进行了基因组学、转录组学、蛋白组学分析。有研究^[48, 49]^显示人表皮生长因子受体2(human epidermal growth factor receptor 2, HER2)的表达改变与微乳头亚型相关，通过原位杂交和IHC检测发现在40%以上的尿路上皮微乳头状癌中存在*HER2*扩增现象，并引起HER2蛋白过表达，生存分析结果表明该类患者较未发生扩增者预后差。还有研究^[50]^发现在晚期尿路上皮微乳头状癌中HER2的胞外结构域中常发生改变。对结直肠肿瘤进行特征基因评估，结果显示在结直肠微乳头状癌中肿瘤蛋白53(tumor protein53, *TP53*)改变的频率较高，微卫星不稳定性和复制错误表型(失配修复蛋白的丢失)的发生率较低^[51]^。通过将微乳头状乳腺癌与其雌激素受体匹配的浸润性导管腺癌比较发现，微乳头状腺癌增殖率高，此外免疫组化检测和芯片杂交分析显示该癌种细胞周期蛋白D1(Cyclin D1)高表达、*MYC*(8q24)扩增^[52]^。对尿路上皮癌中微乳头亚型进行mRNA表达检测，结果显示在该型肿瘤miR-296表达下调，染色质重塑复合物RUVBL1(RuvB-like 1)激活，此遗传学改变可进一步激活Scrib、HMGA1(high mobility group AT-hook 1)和Pin1(peptidyl-prolyl cis-trans isomerase NIMA-interacting 1)介导的致癌通路，促进肿瘤发生发展。进一步对微乳头状尿路上皮癌和常规尿路上皮癌的表达谱差异富集分析发现，在微乳头型肿瘤中细胞周期调控、DNA修复损伤、信号传导通路显著激活^[46]^。

上述结果表明微乳头状腺癌中致癌因子通路可产生不同的激活，癌症相关基因*HER2*、*TP53*变异以及细胞周期调控的异常在微乳头状腺癌中常见。目前针对MPC特征性分子检测主要通过单基因测序、荧光原位杂交技术(fluorescence *in situ* hybridization, FISH)、IHC等方法实施，鉴定结果受研究者研究靶点限制，以致无全面的遗传学分析可供深入指导，因此通过加强MPC的全基因组、转录组、蛋白组研究将为该亚型提供更好的临床指导。

### 2.3 MPC的免疫微环境

随着免疫治疗的开展，有关肿瘤中的免疫微环境的研究日益增多。已有少量研究^[44]^报道了微乳头状腺癌的炎症改变。在胰腺导管腺癌中，微乳头表型和未分化型较其他类型如神经内分泌型、浆液囊腺型等更易出现瘤内大量的中性粒细胞浸润。中性粒细胞在肿瘤中所发挥的作用根据其所处微环境不同而存在差异^[53, 54]^，因此在微乳头成分中浸润的中性粒细胞抗肿瘤功能需进一步明确。在乳腺微乳头状癌中，可见肿瘤浸润淋巴细胞增加，但与乳腺髓质癌中淋巴细胞浸润引起良好预后不同，微乳头状癌中的淋巴细胞多浸润在基质中，且CD8+T细胞Fas及肿瘤细胞FasL表达弱，穿孔素及颗粒酶表达也较少，故而有效细胞毒性T淋巴细胞作用少，此类浸润反而带来了不良的预后^[55]^。综上所述，MPC中炎症微环境可能处于一个抑制状态，通过联合免疫治疗的开展可能使微乳头状腺癌患者受益。

## 3 结语

LMPC作为高级别腺癌之一，与频繁发生转移、淋巴结浸润、疾病复发率增加和总体生存率降低相关，因此早期LMPC积极的治疗方案需要我们进一步完善。同时随着癌症分子和基因组图谱分析技术的进步，精准治疗时代得以到来，鉴于部分致癌因子通路的激活和肿瘤免疫微环境的建立介导了MPC的发生发展，LMPC中特征性因子的鉴定及免疫学特征分析将有助于指导靶向治疗和免疫治疗措施同常规治疗方案的结合，从而提高患者获益。
